# Indirect Inguinal Hernia Causing Testicular Ischemia: A Case Report

**DOI:** 10.7759/cureus.102940

**Published:** 2026-02-04

**Authors:** Hadi Aldarwish, Yousif Alsaeed, Rawad Alrebih, Khatoon Alshaikhmohamed, Abdulaziz Shaikh Mohammed

**Affiliations:** 1 Department of Urology, King Fahad Specialist Hospital, Dammam, SAU; 2 Department of Surgery, Qatif Central Hospital, Qatif, SAU; 3 Department of Surgery, Alexandria University, Alexandria, EGY

**Keywords:** inguinal hernia, orchidopexy, spermatic cord, testicular torsion, testis

## Abstract

Testicular ischemia secondary to an incarcerated inguinal hernia is a rare but serious complication that requires urgent surgical intervention. We report the case of a 31-year-old man who presented to the emergency department with severe left testicular pain, inguinal fullness, and scrotal swelling. Urgent scrotal ultrasonography suggested that the patient had testicular torsion. Intraoperatively, no torsion was identified, and the hernia had extended into the scrotum, causing compression and constriction of the spermatic cord. Furthermore, inguinal hernia repair with mesh and left orchidopexy were performed. Follow-up ultrasonography demonstrated good testicular vascularity. The patient tolerated the procedure well and was discharged in good condition.

## Introduction

Testicular ischemia is most commonly caused by testicular torsion [1]. Moreover, testicular torsion represents a serious and time-critical urological emergency [2]. Although the condition typically affects young men with a bimodal distribution peaking in the perinatal and pubertal periods, the prevalence of testicular torsion in adults is now recognized to be higher than previously appreciated [3,4]. The diagnosis of testicular torsion relies on clinical examination and is supported by radiological imaging such as ultrasonography [5,6]. Once confirmed, urgent surgical intervention within the "golden hours" is essential to restore blood flow and detorse the affected testis [7].

However, in rare cases, testicular ischemia may occur secondary to an indirect inguinal hernia [8]. Although this has been well documented in pediatric patients, a literature review revealed only a limited number of reported cases in adults [9].

In this context, we report a rare case of a 31-year-old man who developed severe testicular pain due to testicular ischemia secondary to an indirect inguinal hernia.

## Case presentation

A 31-year-old man with no known chronic medical conditions presented to the emergency department with severe left testicular pain and scrotal swelling that began approximately three hours before presentation. His medical history was notable for right inguinal hernia repair 15 years ago and pilonidal sinus excision in 2014.

The patient experienced sudden-onset left scrotal pain, described as draping in nature, and radiating to the left inguinal area. The pain was not associated with nausea or vomiting but was accompanied by marked scrotal swelling and tenderness. The patient rated the severity of pain as 10 out of 10.

The onset of swelling occurred after lifting heavy objects. The patient reported a 15-year history of intermittent swelling in the left inguinal region. He denied any history of fever, urinary symptoms, similar episodes, abdominal pain, nausea, vomiting, or constipation. On examination, he was hemodynamically stable.

An abdominal examination revealed a soft, non-distended, and non-tender abdomen. Nonreducible fullness was observed in the left inguinal region. Genital examination revealed marked swelling and tenderness of the left scrotum. Examination of the left testicle was limited due to swelling, while the position and consistency of the right testicle were normal. Key laboratory results are summarized in Table 1.

**Table 1:** Laboratory results of the patient (other parameters were unremarkable).

Parameter	Results	Normal ranges
White Blood Cell Count	5.16 x10^9/L	4.5 to 11.0 ×10^9/L
Hemoglobin	13.7 g/dL	13 to 17 g/dL
Platelet Count	223 x10^9/L	150-430 x10^9/L

Relevant investigations

Scrotal ultrasonography revealed a heterogeneously hypoechoic left testicle with absent vascularity on color Doppler. The left testicle measured 3.4 x 3.1 cm, whereas the right testicle measured 4.2 x 2.3 cm. An associated small right hydrocele was noted with internal echogenic debris. The right testicle exhibited homogenous echogenicity with preserved vascularity. The left hemiscrotum contained echogenic material resembling subcutaneous fat, with fine internal echoes, likely representing a large fat-containing inguinal hernia. No definite bowel loops were identified.

Impression

**Figure 1:**
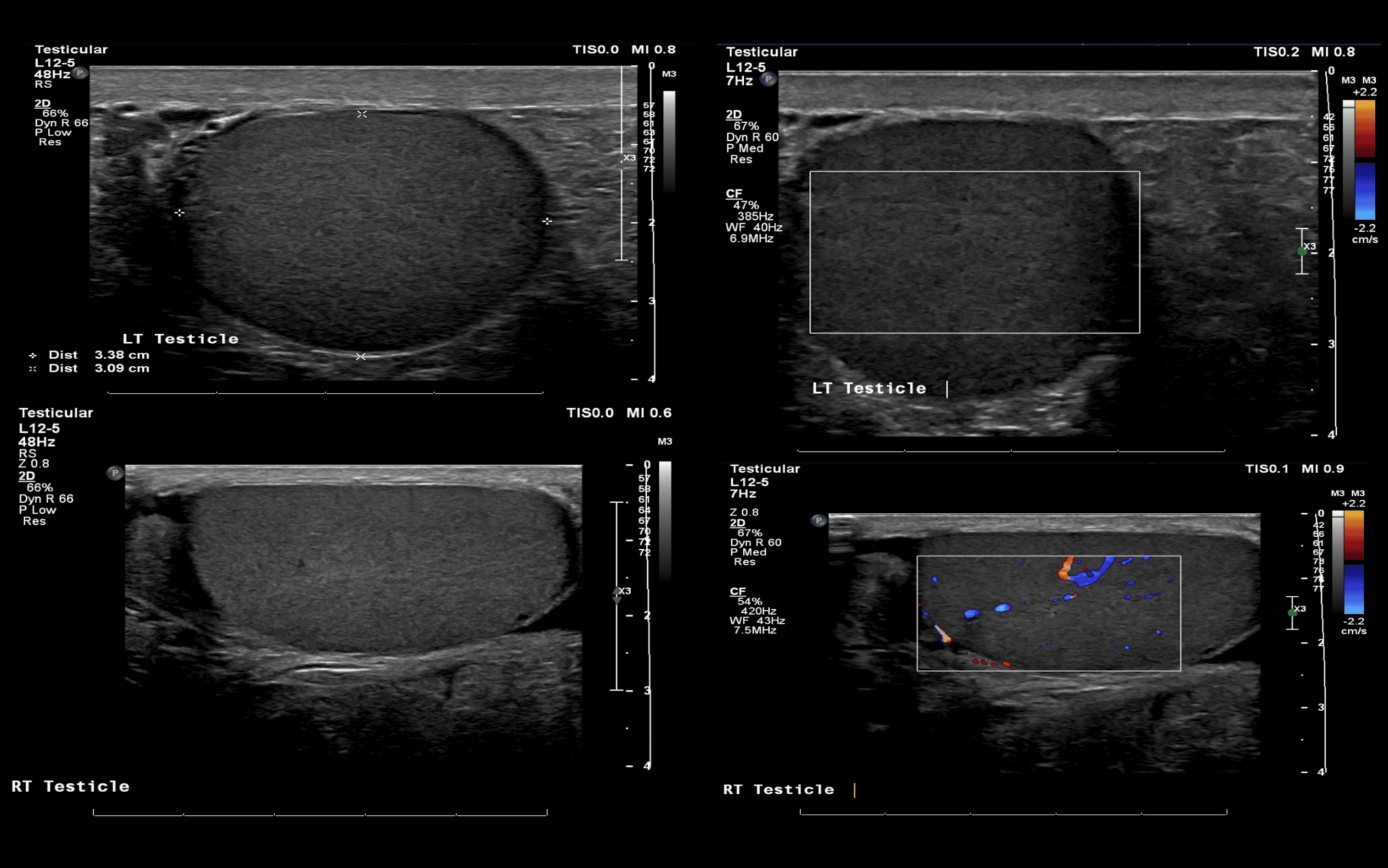
Scrotal ultrasound showing no vascularity in the left testis.

Findings described above are highly suggestive of left testicular torsion for urgent urology consultation (Figure 1).

**Figure 2:**
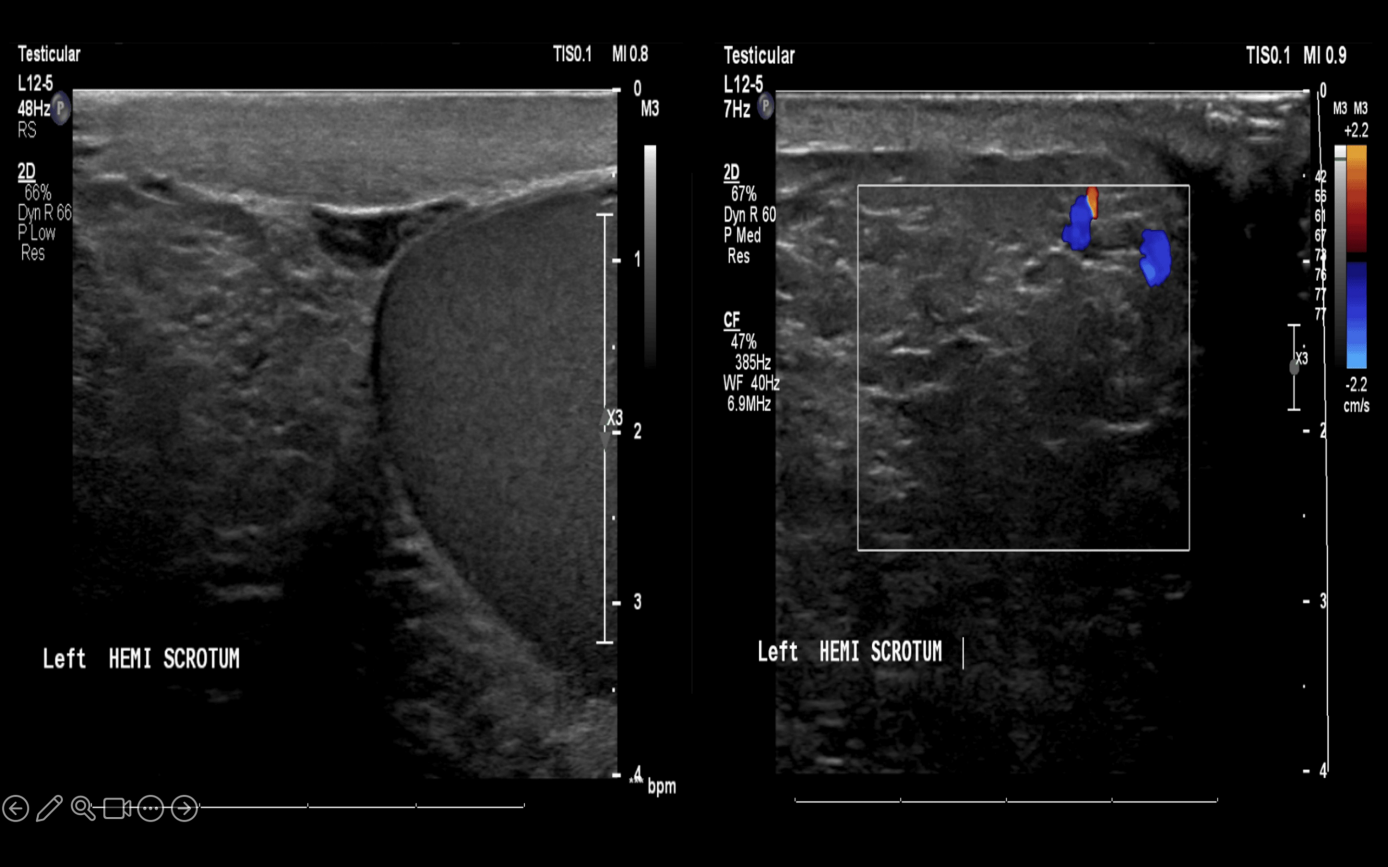
Ultrasound showing left hemiscrotum occupied by echogenicity

The left hemiscrotum contained an echogenic material similar to subcutaneous fat, with fine internal echoes, likely representing a large fat-containing inguinal hernia, with no definite bowel loop content identified. Further evaluation with computed tomography was warranted (Figure 2).

Surgical events

The findings were discussed with the patient and his family. At that time, we determined that priority should be given to addressing the suspected torsion by urology, with general surgery to follow thereafter. The patient and his family provided informed consent to participate in this study. 

The procedure was performed under general anesthesia. The patient was prepared and draped in a sterile manner. Scrotal examination and manual attempts at hernia reduction were unsuccessful, prompting a 4 cm left lateral scrotal incision. Blunt and sharp dissection through the darkened muscle and fascia revealed a large hernial sac. The left testis was adherent to the hernial sac but remained viable with no evidence of torsion. Attempts to separate the testis from the sac were unsuccessful; therefore, a general surgical team was consulted. Both general surgery and urology specialists attempted separation, and the peritoneum was opened to expose the omentum. Testes were successfully isolated and confirmed to be viable. The general surgical team decided to proceed with the inguinal hernia repair. 

An oblique open hernial incision was made above the anatomical landmark of the inguinal ligament. The sheath and external inguinal ring were identified and opened. The spermatic cord was visualized and separated from the floor of the hernia. The vas deferens was identified, although the separation from the hernial sac proved challenging. The sac was opened, revealing an incarcerated greater omentum extending into the scrotum. As the omentum could not initially be reduced, partial resection was performed using surgical ligation, after which the remaining omentum was successfully removed from the scrotum. The hernial sac was firmly adherent to the vas deferens and could not be separated; therefore, the sac was ligated with 3-0 absorbable sutures and excised. The distal portion of the sac was excised and opened to prevent seroma formation. A surgical mesh was placed for reinforcement. The fascial sheath was closed using size 1 absorbable sutures, and the skin was closed using surgical clips. 

After completing the repair, left orchiopexy was performed using two 2-0 silk sutures, one inferior and one lateral, to secure the testis in its normal position. The fascia was closed continuously with 3-0 Vicryl, and the skin was closed with simple interrupted 2-0 Monocryl sutures. A pressure dressing was applied; the patient tolerated the procedure well, being transferred to the recovery unit in stable condition.

Post-operation follow-up

**Figure 3:**
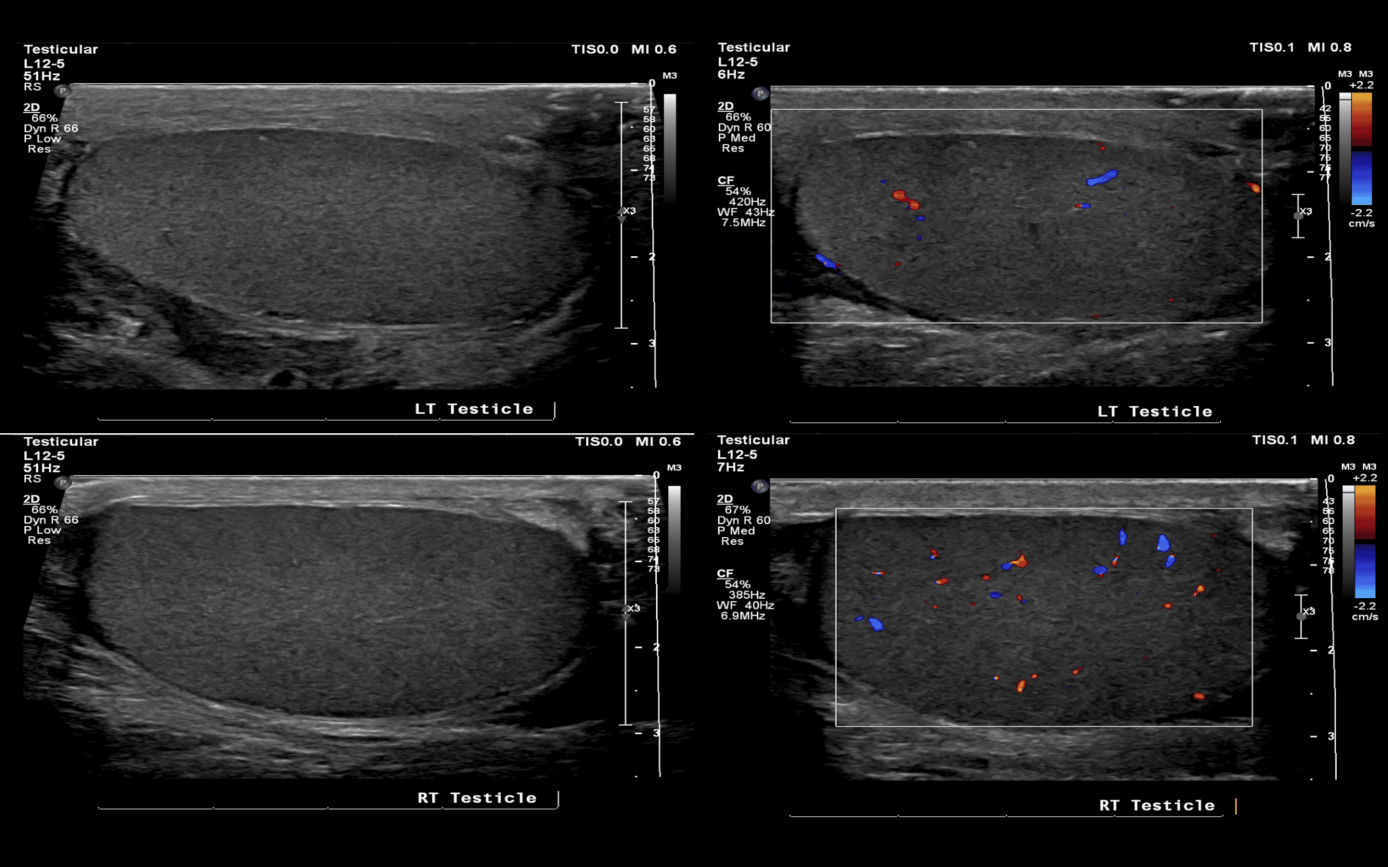
Follow-up ultrasound showing testicular vascularity to be preserved bilaterally

Two weeks postoperatively, the patient returned to the outpatient clinic for follow-up with both urology and general surgery. Ultrasonography demonstrated that both testes were normal in size and echotexture. Vascularity was preserved bilaterally on color Doppler, although it appeared slightly reduced on the left, likely due to technical factors. However, focal testicular lesions were not observed. The epididymides were bilaterally unremarkable. A left-sided varicocele was observed along with a small right hydrocele (Figure 3).

The histopathological report of the omentum revealed fatty tissue with congested blood vessels and a focal area of hemorrhage; otherwise, no significant pathology was detected. Histopathology of the hernial sac demonstrated vascularized fibro-fatty connective tissue partially lined with mesothelial cells, consistent with the hernial sac.

Clinically, the patient was doing well. The wound had healed with only mild swelling and numbness in the left thigh. The patient was reassured and scheduled for a follow-up appointment in one month. 

## Discussion

Inguinal hernia is a common condition, with prevalence among men ranging from 0.6% to 25.2%, depending on age and population demographics [10]. Although rare, incarcerated inguinal hernia leading to testicular ischemia is a serious complication that requires prompt recognition and timely intervention [11].

Although inguinal hernias are frequently encountered in surgical practice, their potential to compromise the testicular perfusion remains unpredictable [9]. Moreover, a review of the literature indicates that such cases are more commonly reported in pediatric patients than in adults [12].

Turgut et al. [13] reported that an inguinal hernia may impair testicular blood flow due to intermittent mechanical compression of the spermatic cord (funiculus spermaticus) within the inguinal canal. Additionally, in some cases, inappropriate attempts at manual hernia reduction may contribute to testicular ischemia by compressing vascular structures with the hernia contents [14].

In the present case, the hernia was not reducible, with the patient’s primary complaint being testicular pain. An incarcerated inguinal hernia can increase intracanalicular pressure, thereby reducing testicular blood flow [9]. Our patient experienced mechanical compression of the spermatic cord, resulting in testicular ischemia, a condition that may progress to infarction if surgical intervention is delayed.

This case emphasizes the importance of considering testicular ischemia in adult men presenting with incarcerated inguinal hernias. Anatomical variations such as a narrow internal ring or a bulky, fat-containing hernia sac, as observed in this case, may predispose patients to such complications. Surgical exploration is both diagnostic and therapeutic.

## Conclusions

We report a rare case of testicular ischemia secondary to an incarcerated inguinal hernia in an adult male patient. In this case, urgent surgical intervention was both diagnostic and therapeutic, playing a crucial role in preventing permanent testicular damage. Urologists and general surgeons should maintain a high index of suspicion for testicular ischemia when signs of testicular involvement are present in patients with an inguinal hernia. Additional studies are warranted to better understand the pathophysiology of this rare but serious complication.
